# ST913-IVa-t991 Methicillin-Resistant *Staphylococcus aureus* among Pediatric Patients, Israel

**DOI:** 10.3201/eid3008.230981

**Published:** 2024-08

**Authors:** Moti Baum, Einav Anuka, Maya Davidovich-Cohen, Assaf Rokney

**Affiliations:** Ministry of Health, Jerusalem, Israel

**Keywords:** methicillin-resistant Staphylococcus aureus, MRSA, antimicrobial resistance, local clone, whole-genome sequencing, pediatric clone, Israel, bacteria

## Abstract

In Israel, prevalence of sequence type 913, staphylococcal cassette chromosome *mec*IVa, *spa* type t991 methicillin-resistant *Staphylococcus aureus* lineage has surged among pediatric populations, predominantly in Arab and Orthodox Jewish communities. Antimicrobial resistance patterns vary by demographics. This lineage's spread and microevolution in the Middle East underscore the need for ongoing surveillance.

In 2010, a new methicillin-resistant *Staphylococcus aureus* (MRSA) clone, belonging to the clonal complex (CC) 913, Panton-Valentine leukocidin (PVL)–negative, staphylococcal cassette chromosome *mec* type IV, was isolated from Bedouin children in Israel (*1*). In 2012, isolates of CC913 were further analyzed, and their *spa* type was revealed as t991. Four t991 isolates were identified in hospitals across Israel, indicating the spread of the clone to communities beyond the Bedouin population in southern Israel (*2*). In 2015, a total of 12 t991 isolates were obtained from 280 patients (*3*), and in 2019, a total of 6 t991 isolates were obtained from 112 patients (*4*), mainly from children.

Since 2015, MRSA isolates of *spa* type t991 have emerged to become one of the main lineages in hospitals and health maintenance organizations in Israel. However, despite its significance, comprehensive characterization of *spa* type t991 clone is lacking. We explore its genomic context and antibiotic profile in this study.

## The Study

**Figure 1:**
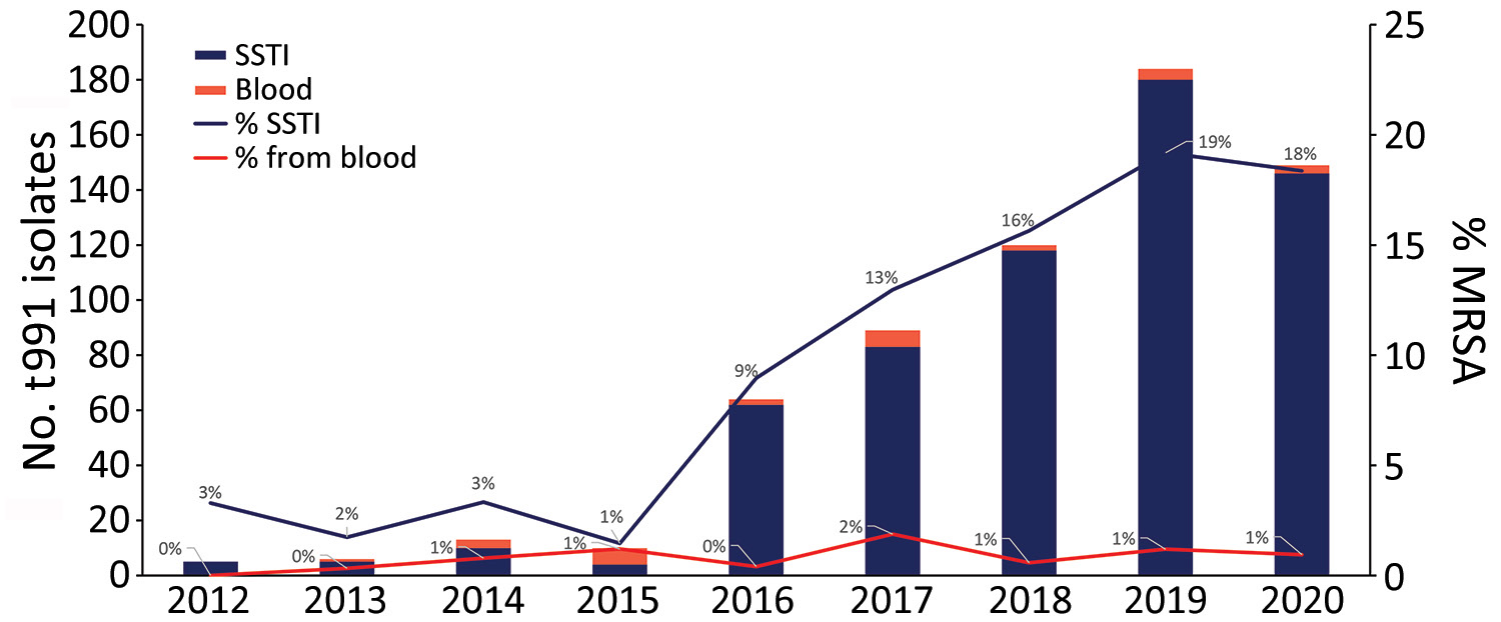
t991 MRSA isolates isolated from blood and SSTIs among patients in Israel during 2012–2020. Left y-axis represents number of t991 isolates isolated and right y-axis represents the relative part from total MRSA SSTI or blood isolates. MRSA, methicillin-resistant *Staphylococcus aureus*; SSTI, skin and soft tissue infection.

 During 2012–2020, the *S. aureus* national reference laboratory of Israel received a total of 4,646 MRSA isolates, obtained from skin and soft tissue infections (SSTIs), that were classified into 284 different *spa* types. Types t002, t008, and t032 were the most prevalent; t002 comprised 25% of total MRSA SSTI isolates, t008 comprised 15%, and t032, 5%. During that period, the proportion of t991 MRSA gradually increased to 13% of total MRSA SSTI isolates, whereas the leading *spa* types in MRSA SSTIs (t002 and t008) remained stable (Appendix Figure 1). During that period, 689 *S. aureus* samples of *spa* type t991, *mecA*-positive, PVL-negative, were received at the *S. aureus* national reference laboratory (Appendix). Most of the samples (406, 70%) were isolated from SSTIs; 66% isolates were from patients <5 years of age (p = 0.0001), whereas 4% were isolated from patients >60 years of age. In addition, most patients resided in localities associated with Arab and Orthodox Jewish populations (*5*). The number of t991 MRSA isolates from SSTI and blood increased dramatically, from 5 in 2012 to 180 in 2019 and 146 in 2020 (https://microreact.org/project/r4dFwJGXudh3gfyWtE1f87-t991final) (Figure 1).

**Figure 2:**
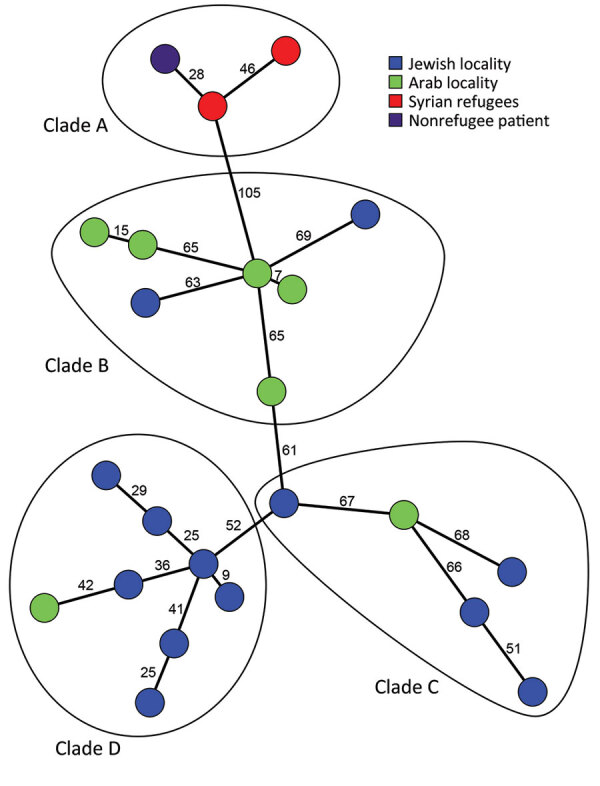
Phylogenetic relationships between 23 t991 MRSA genomes isolated in Israel and Germany. The figure shows a minimum spanning tree, created in Bionumerics software (https://www.bionumerics.com), based on 3,904 wgMLST allele IDs of sequenced t991 MRSA isolates. Each node represents an isolate; numbers along branches connecting nodes indicate the numbers of allelic differences between isolates. The isolates are further divided into 4 clades (A–D). MRSA, methicillin-resistant *Staphylococcus aureus;* wgMLST whole-genome multilocus sequence typing.

We conducted whole-genome sequencing on 20 t991 MRSA isolates that were selected (Appendix Table), along with 3 t991 MRSA isolates from Germany (*6*,*7*). The isolates clustered into 4 separate clades (Figure 2). Clade A consisted of the 3 t991 isolates from Germany and is 130 whole-genome multilocus sequence typing (wgMLST) alleles distant from the first isolate of clade B, which consisted of 7 isolates from patients who lived in the Negev and were admitted to the same hospital. Clade C consisted of 5 isolates from patients residing in the Jerusalem district. Clade D consisted of 8 isolates, 7 of which were from Orthodox Jewish patients.

**Figure 3:**
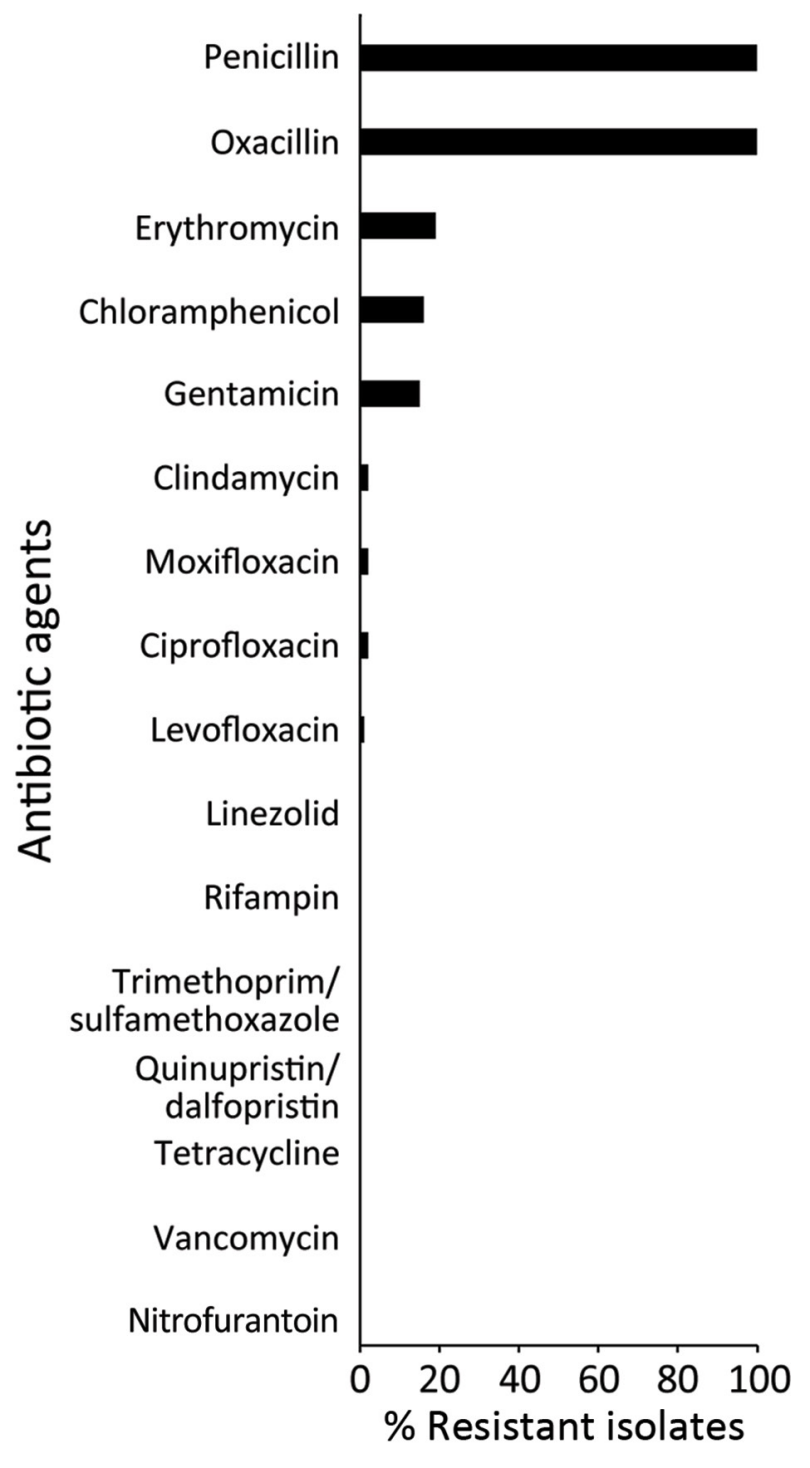
Percentage of resistant isolates to antibacterial agents among 116 t991 MRSA isolates from Israel tested for antimicrobial susceptibility using the broth microdilution method. MRSA, methicillin-resistant *Staphylococcus aureus.*

We tested 116 t991 MRSA isolates for phenotypic susceptibility by using the broth microdilution method (Figure 3). Isolates from patients living in Arab localities were more resistant to erythromycin and chloramphenicol, whereas those isolated from patients living in Jewish localities showed higher resistance to gentamicin, ciprofloxacin, levofloxacin, and moxifloxacin (Appendix Figure 2). That tendency was statistically significant for chloramphenicol (p = 0.01) and gentamicin (p = 0.01).

**Figure 4:**
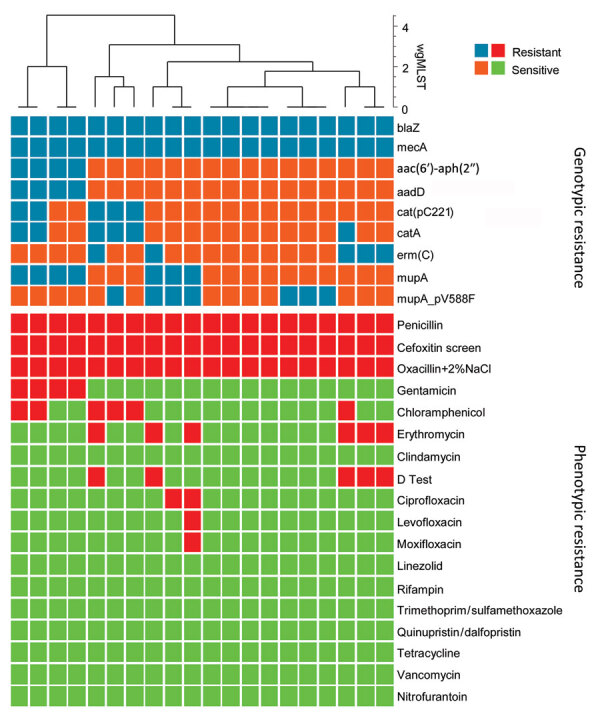
Comparison of genotypic and phenotypic resistance patterns of 20 t991 MRSA isolates from Israel tested using whole-genome sequencing. Blue tiles represent presence of resistance gene and orange tiles absence of resistance gene; red tiles represent antimicrobial resistance and green tiles antimicrobial sensitivity. Clustering is based on wgMLST data and generated by BioNumerics software (https://www.bionumerics.com). wgMLST, whole-genome multilocus sequence typing.

We found 9 antimicrobial resistance (AMR) determinants, 8 AMR genes and 1 point mutation, among the 20 t991 MRSA sequences (Figure 4). Overall, correlation between genotype prediction based on WGS and phenotypic AMR was 99% with a sensitivity of 94% and specificity of 100%. All discrepancies were associated with an absence of resistance determinant among phenotypically resistant isolates. No data for quinolone resistance genes or mutational resistance were predicted by the BioNumerics (https://www.bionumerics.com) or AMRFinder (https://github.com/ncbi/amr/releases/tag/amrfinder_v3.10.21) algorithms. In addition, we did not test phenotypic resistance against mupirocin.

We compared virulence profiles of 20 WGS t991 isolates with 3 t991 isolates from Germany, community-acquired MRSA USA300, and USA400 (*8*) as a reference using the functional genotyping tool of Bionumerics version 8.0. The main difference in virulence gene profile is reflected in the group of genes associated with adherence (Appendix Figure 3). In addition, virulence profiles can be grouped into 6 patterns on the basis of the presence of specific adherence factor genes in the genomes (Appendix Figure 4). We found no correlation between virulence profile and AMR, age, sex, residence location, or association with 1 of the genomic clades.

Next, to assess the genetic relationship of t991 isolates to other MRSA strains circulating worldwide, we created GrapeTree on the pubMLST site (https://pubmlst.org/organisms/staphylococcus-aureus) based on wgMLST data of representative local MRSA t991 strain (SA14675) along with 37,883 *S. aureus* global isolates (Appendix Figure 5) (*9*). The closest node to strain SA14675 is at a distance of 74 wgMLST alleles and is composed of 7 isolates. The next closest node is at a distance of 1,486 wgMLST alleles and composed of isolates that belong mainly to CC1.

## Conclusions

Most t991 cases were isolated from young patients who live in strictly Orthodox and Arab localities. A possible explanation for this phenomenon is a similar lifestyle of the 2 sectors, characterized by overcrowding and large families. Regarding the evolution of this clone and its spread into the population, this strain appears to have evolved by multiple different genetic events. This assumption is supported by several findings. First, t991 MRSA isolates demonstrated classification into 4 distinct clades on the basis of geographic location and sectoral association; we noted genetic variation and weak clonality evident from the considerable distances between nodes, even within the same clade. Second, antibiotic resistance patterns vary between isolates obtained from patients who live in Jewish and Arab localities (Appendix Figure 2). Finally, *spa* type t991 composition is very short; it consists of 3 repeats (07-33-23) and can be formed as a result of genetic rearrangement of numerous MRSA strains harboring longer *spa* type repeats in which the repeats of *spa* type t991 from a part of their repeat succession. Worldwide phylogenetic analysis indicates that t991 MRSA stands out as a distinct emerging lineage because it appears considerably distant from most strains included in the GrapeTree (Appendix Figure 5).

Phenotypic AMR data for global isolates were available for 2 isolates (*7*,*10*). One isolate obtained in Kuwait was resistant to erythromycin, clindamycin, trimethoprim, and fusidic acid (*10*), and the other was isolated in Germany from a refugee from Syria (*7*) and was resistant to erythromycin, clindamycin, and tetracycline. Out of the 116 tested t991 isolates, none showed resistance to tetracycline by antimicrobial susceptibility. For 2 isolates, we observed phenotypic resistance for erythromycin and chloramphenicol without prediction of AMR determinant. Close inspection of those isolates revealed that were actually positive for *ermC* and *cat*, and their sequences were fragmented into multiple contigs. Consistent with previous publications (*10*–*12*), all isolates tested, except for the strain from the Syria refugee (*7*), were positive for *eta*, a toxin responsible for skin infections seen mainly among young patients (*3*,*4*,*7*). Those findings are in accordance with the observation that t991 MRSA is predominantly isolated from children.

In conclusion, our study shows the emergence of t991 MRSA in Israel. These strains affect mainly pediatric populations, and a geographic distribution is limited mainly to the Middle East. The epidemiologic and genomic information our research provides will assist further investigation on the origin and dissemination of this clone.

Appendix Additional information about ST913-IVa-t991 methicillin-resistant *Staphylococcus aureus* clones in pediatric patients, Israel.
