# Supplementary material for: ST913-IVa-t991 Methicillin-Resistant Staphylococcus aureus among Pediatric Patients, Israel
Source: Emerg Infect Dis. 2024 Aug;30(8):1714–8. doi: 10.3201/eid3008.230981 (PMC11286069; doi:10.3201/eid3008.230981)
Supplement: Appendix — Additional information about ST913-IVa-t991 methicillin-resistant Staphylococcus aureus clones in pediatric patients, Israel. [file 23-0981-Techapp-s1.pdf]

Article DOI: http://doi.org/10.3201/eid3008.230981

EID cannot ensure accessibility for supplementary materials supplied by authors. Readers who have difficulty accessing supplementary content should contact the authors for assistance.

ST913-IVa-t991 Methicillin-Resistant *Staphylococcus aureus* among Pediatric Patients, Israel

Appendix

Materials and Methods

Bacterial isolates, media and lysate preparation – *S. aureus* isolates of *spa* type t991 originated mainly from SSTI between 2012 to 2020. The samples were submitted to the National *S. aureus* reference laboratory (NRL) from clinical labs located across the State of Israel for bacteriological characterization. In Israel clinical labs are required to send MRSA and MSSA isolates from bacteremia, along with MRSA from wound infections, to the national center for further analysis and storage in our strain bank. All strains are sent along with demographic data and isolation source information. Strains NCTC 13552 and NCTC 14245 were used as positive control in *mecA*, *mecC* and PVL real time PCR reaction. Strain SA104 (*mecA* and PVL positive, *spa* type t008) was used as a positive control in *spa* PCR. ATCC strain 29213 was used an Internal quality control in the broth microdilution assay. All strains were cultured at 37°C for 16-24 hours.

The strains were cultured on CHROMagar MRSA/MSSA plates (DD066, hylabs, Israel) and a single colony was transferred to Nutrient Agar (PD040, hylabs, Israel). Lysis of bacterial cells was performed by suspending a single colony in 100µl lysis buffer (lysozyme 50.8 units; lysostaphin 2.7 units; TRIS 0.1M pH=8; EDTA 0.01M; DDW to a final volume of 100 µl) and incubation at 37 °C for 30 min following 10 min of boiling. The lysates were diluted 1:40 and 2 µl of the dilution was used as a template in real time and PCR reactions.

Real time PCR and *spa* PCR: multiplex real time PCR for the simultaneous detection of *mecA*, *mecC*, PVL and *nuc* gene, which serve as an internal amplification control, was performed

as previously described by Pichon et al (PMID: 22687894), Fosheim et al (PMID: 21697325)and CDC document No ARC.TE.C.0055. In the NRL *spa* type analysis is performed on all MRSA and PVL positive MSSA isolates Molecular typing by *spa* type was performed as previously described by Kahl et al (PMID: 15635028). Analysis of *spa* sequences was performed using BioNumerics 8.0 software (Applied Maths, Belgium).

Antimicrobial susceptibility testing: antimicrobial susceptibility testing was performed using the broth microdilution method. Sensititre susceptibility plates (gram positive GPALL1F AST Plate) was used according to the manufacturer's instructions. Briefly, ~ 5 colonies were suspended in ddw to a turbidity of 0.5 McFarland. Then, 10 µl of the suspension were transferred into 11 ml of cation-adjusted Mueller-Hinton broth (cat. Number T3462). The plate was inoculated using the Sensititre AutoInoculator / AIM. Following 24 hours incubation, results were read using the VIZION platform (Sensititre). Minimum inhibitory concentrations (MIC) were determined according to CLSI guidelines (M100 2020). Correlation, sensitivity and specificity calculation were performed as previously described by Rokney et al (PMID: 32903472).

Genomic DNA extraction, WGS analysis and Bioinformatics analysis Genomic DNA extraction was performed by using the QIAsymphony platform (QIAGEN, Hombrechtikon, Switzerland). DNA library was prepared using Nextera XT kit (Illumina Inc. San Diego, CA, USA). *De novo* assembly by SPAdes and whole genome MLST (wgMLST) was performed on the BioNumerics 8.0 software (Applied Maths, Belgium) by using the default settings. Genomic comparison were visualized using minimum spanning tree (MST) based on 3,904 wgMLST allele IDs by using the BioNumerics 8.0, with alleles difference annotated on the branches. Resistance genes and virulence factor detection was carried out on the BioNumerics 8.0 by using the *S. aureus* functional genotyping tool. In addition, antimicrobial resistance genes presence was predicted by the AMRfinder software.

Grapetree creations GrapeTree was created on the pubMLST site. In short, the genome collection database was filtered by sequence bin (total length > 2.5 Mbp) and GrapTree was created using cgMLST of 37,883 *Staphylococcus aureus* isolates using the default settings and colored by clonal complex (MLST).

Statistical analysis: Statistical analyses were performed with GraphPad (https://www.graphpad.com). Data was considered as statistically significant if $p < 0.05$.

The following genomes were used for WGS analysis comparison: t991 isolate from Syrian refugee in Germany in 2018 (PMID: 34785377). T991 isolates from Syrian refugee and non-refugee patient in Germany in 2015 and 2016 respectively (PMID: 29851962). Strain SA14675 was deposited in the pubMLST database under the ID 36889.

Appendix Table. Demographic characteristics and antibiotic resistance profiles of 20 MRSA strains analyzed by whole-genome sequencing

#	Ref lab	Sector	Sample Collection Date	Originator	Residence Place	AST Pattern	WGS	CHL Int	Cip Int	Clinda Int	Dtest 1 Int	Dtest 2 Int	Ery Int	Genta Int	Levo Int	Moxi Int
1	SA09749	Orthodox	7/27/2017	HMO	Modi'in Illit	A	Orthodox_R to Chloramphenicol only	**R**	S	S	S	S	S	S	S	S
2	SA10528	Arab	11/17/2017	Hospital	Unknown	A	Arab_R to Chloramphenicol only	**R**	S	S	S	S	S	S	S	S
3	SA08727	Orthodox	2/28/2017	Hospital	Modi'in Illit	B	Jewish_R only to Erythromycin	S	S	S	**R**	**R**	**R**	S	S	S
4	SA11866	Orthodox	5/23/2018	HMO	Elazar	B	Orthodox_R only to Erythromycin	S	S	S	**R**	**R**	**R**	S	S	S
5	SA13166	Arab	1/16/2019	Hospital	Rahat	B	Arab_R only to Erythromycin	S	S	S	**R**	**R**	**R**	S	S	S
6	SA08765	Arab	3/5/2017	Hospital	Jerusalem	C	Arab_R to Chloramphenicol and Gentamicin	**R**	S	S	S	S	S	**R**	S	S
7	SA10010	Orthodox	8/27/2017	HMO	Beit Shemesh	C	Orthodox_R to Chloramphenicol and Gentamicin	**R**	S	S	S	S	S	**R**	S	S
8	SA09965	Arab	8/17/2017	Hospital	Huzail tribe	D	Arab_First strain R to Chloramphenicol and Erythromycin	**R**	S	S	**R**	**R**	**R**	S	S	S
9	SA12099	Arab	7/25/2018	Hospital	Rahat	D	Arab_Last strain R to Chloramphenicol and Erythromycin	**R**	S	S	**R**	**R**	**R**	S	S	S
10	SA08479	Orthodox	12/21/2016	HMO	Modi'in Illit	E	Orthodox_First strain S to all antibioticss tested	S	S	S	S	S	S	S	S	S
11	SA08650	Arab	2/16/2017	Hospital	Modi'in Illit	E	Arab_First strain S to all antibioticss tested	S	S	S	S	S	S	S	S	S
12	SA09589	Jewish	7/11/2017	Hospital	Jerusalem	E	Jewishl_First strain S to all antibiotics tested	S	S	S	S	S	S	S	S	S
13	SA13843	Orthodox	4/29/2019	HMO	Jerusalem	E	Orthodox_Last strain S to all antibioticss tested	S	S	S	S	S	S	S	S	S
14	SA14456	Arab	8/14/2019	Hospital	Umm Batin	E	Arab_Last strain S to all antibioticss tested	S	S	S	S	S	S	S	S	S
15	SA14675	Jewish	9/12/2019	Hospital	Shlomit	E	Jewishl_Last strain S to all antibiotics tested	S	S	S	S	S	S	S	S	S
16	SA11087	Orthodox	1/17/2018	HMO	Bnei Brak	F	Middle strain R to Gentamicin	S	S	S	S	S	S	**R**	S	S
17	SA12245	Orthodox	7/19/2018	HMO	Tel Sheva	F	Last strain R to Gentamicin	S	S	S	S	S	S	**R**	S	S
18	SA07499	Orthodox	8/21/2016	HMO	Beitar Illit	H	First strain from HMO	S	**R**	S	S	S	**R**	S	**R**	**R**
19	SA09727	Orthodox	2/5/2017	HMO	Jerusalem	I	R to Ciprofloxacin only	S	R	S	S	S	S	S	S	S
20	SA06277	Jewish	3/13/2016	Hospital	Beersheba	L	First strain from Hospital	S	R	S	S	S	S	S	R	S

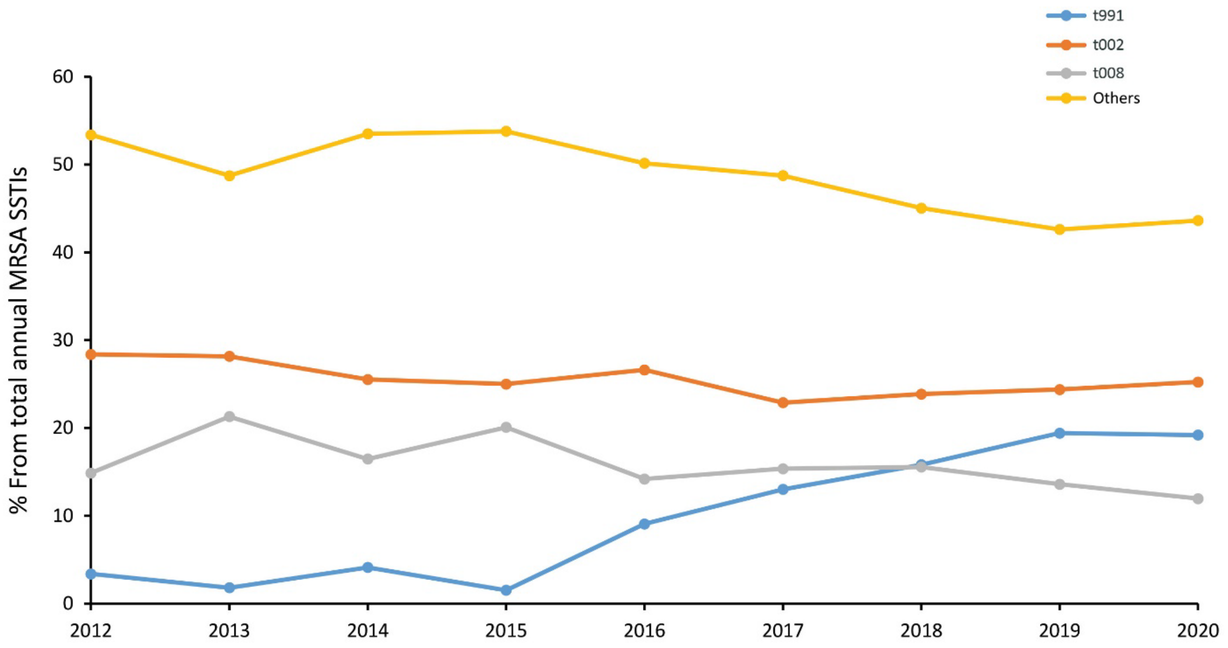

Appendix Figure 1. Prevalence comparison of spa types t991, t002, t008 and all other spa types among clinical MRSA isolates isolated from SSTIs between 2012–2020.

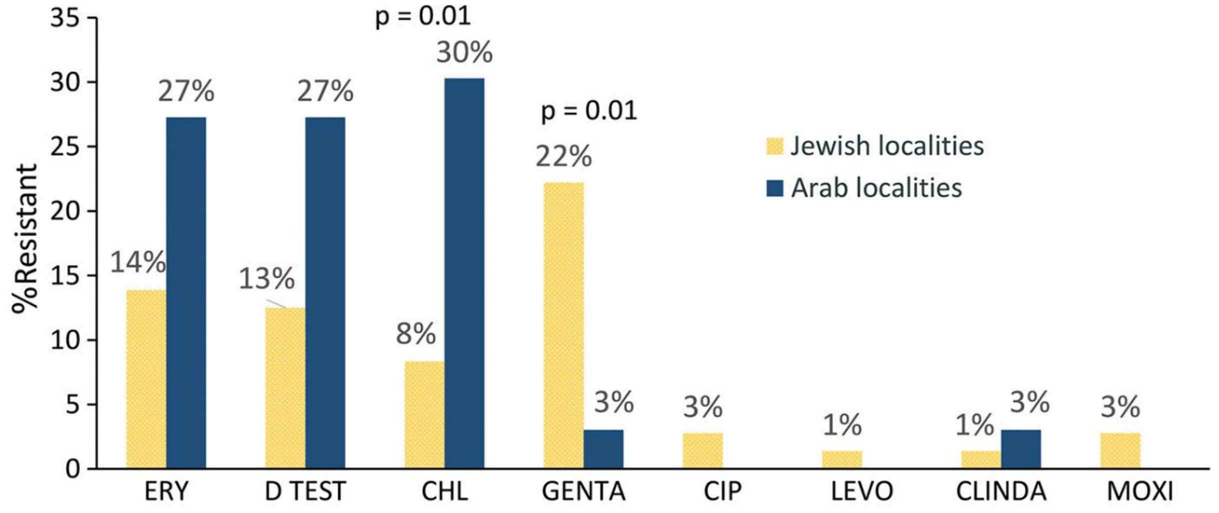

Appendix Figure 2. AMR pattern of t991 isolates, isolated from patients who live in Jewish and Arab localities.

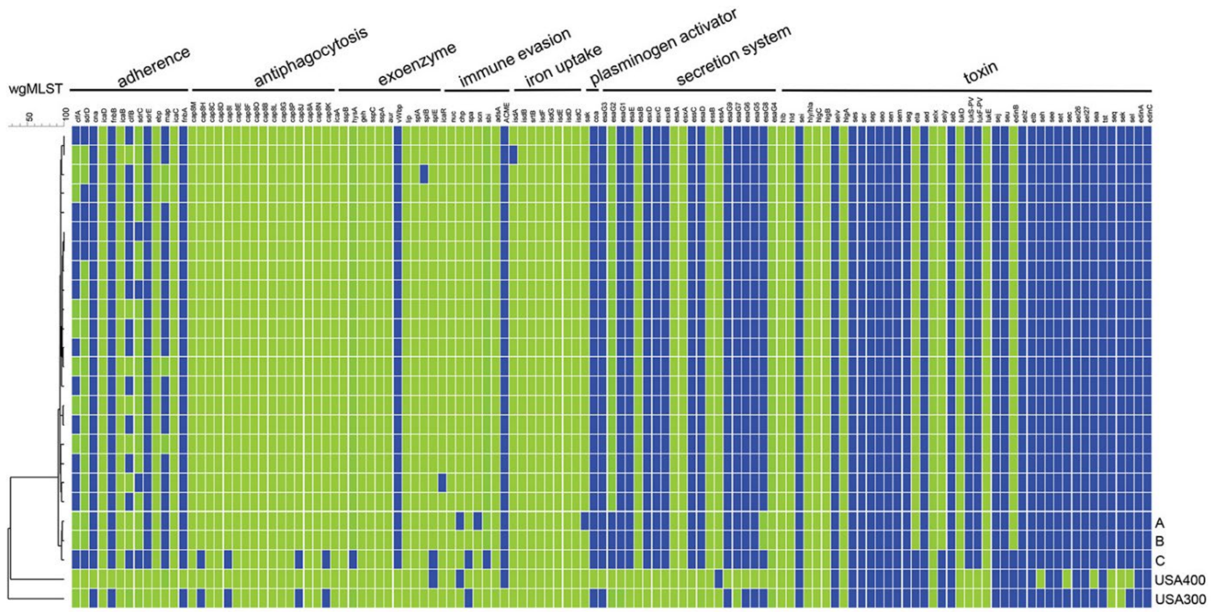

Appendix Figure 3. Comparison of virulence factors profile of t991 MRSA isolates, isolated in Israel and Germany alongside USA300 and USA400 strains. Virulence factors analysis was performed using BioNumerics *S. aureus* functional genotyping tool. Presence or absence of gene is represented by green or blue tile respectively. The dendrogram on the left is based on wgMLST data. A – strain 012 isolated from Syrian refugee, B – strain 073 isolated from non-refugee patient, C – this strain was isolated in Germany from refugee.

	Virulence Pattern	icaB	icaC	icaD	ebp	sdrC	sdrD	clfA	clfB	map	sdrE	cna	fnbB	fnbA
SA08479	A	<+>	<+>	<+>	<+>	<->	<->	<->	<->	<->	<->	<->	<->	<->
t991 isolated from refugee in Germany	A	<+>	<+>	<+>	<+>	<->	<->	<->	<->	<->	<->	<->	<->	<->
SA14456	B	<+>	<+>	<+>	<+>	<+>	<->	<->	<->	<->	<->	<->	<->	<->
SA08650	B	<+>	<+>	<+>	<+>	<+>	<->	<->	<->	<->	<->	<->	<->	<->
SA09749	B	<+>	<+>	<+>	<+>	<+>	<->	<->	<->	<->	<->	<->	<->	<->
SA09965	C	<+>	<+>	<+>	<+>	<+>	<+>	<->	<->	<->	<->	<->	<->	<->
SA07499	C	<+>	<+>	<+>	<+>	<+>	<+>	<->	<->	<->	<->	<->	<->	<->
SA11866	C	<+>	<+>	<+>	<+>	<+>	<+>	<->	<->	<->	<->	<->	<->	<->
SA13843	C	<+>	<+>	<+>	<+>	<+>	<+>	<->	<->	<->	<->	<->	<->	<->
SA10010	C	<+>	<+>	<+>	<+>	<+>	<+>	<->	<->	<->	<->	<->	<->	<->
SA12245	C	<+>	<+>	<+>	<+>	<+>	<+>	<->	<->	<->	<->	<->	<->	<->
SA10528	D	<+>	<+>	<+>	<+>	<+>	<+>	<+>	<+>	<->	<->	<->	<->	<->
SA12099	D	<+>	<+>	<+>	<+>	<+>	<+>	<+>	<+>	<->	<->	<->	<->	<->
SA08727	D	<+>	<+>	<+>	<+>	<+>	<+>	<+>	<+>	<->	<->	<->	<->	<->
Strain 012 from Syrian refugee	D	<+>	<+>	<+>	<+>	<+>	<+>	<+>	<+>	<->	<->	<->	<->	<->
Strain 073 from non-refugee patient	D	<+>	<+>	<+>	<+>	<+>	<+>	<+>	<+>	<->	<->	<->	<->	<->
SA14675	E	<+>	<+>	<+>	<+>	<+>	<+>	<+>	<+>	<+>	<->	<->	<->	<->
SA08765	E	<+>	<+>	<+>	<+>	<+>	<+>	<+>	<+>	<+>	<->	<->	<->	<->
SA09589	F	<+>	<+>	<+>	<+>	<+>	<+>	<+>	<->	<+>	<->	<->	<->	<->
SA06277	F	<+>	<+>	<+>	<+>	<+>	<->	<+>	<->	<+>	<->	<->	<->	<->
SA09727	F	<+>	<+>	<+>	<+>	<->	<+>	<->	<->	<->	<->	<->	<->	<->
SA13166	F	<+>	<+>	<+>	<+>	<+>	<+>	<+>	<->	<->	<->	<->	<->	<->
SA11087	F	<+>	<+>	<+>	<+>	<->	<+>	<->	<+>	<->	<->	<->	<->	<->
USA300 (t991 comparison)	USA300	<+>	<+>	<+>	<+>	<+>	<+>	<+>	<+>	<+>	<+>	<->	<->	<->
USA400 (t991 comparison)	USA400	<+>	<+>	<+>	<+>	<+>	<+>	<+>	<+>	<+>	<+>	<+>	<+>	<+>

Appendix Figure 4. Comparison of virulence pattern as predicted by BioNumerics.

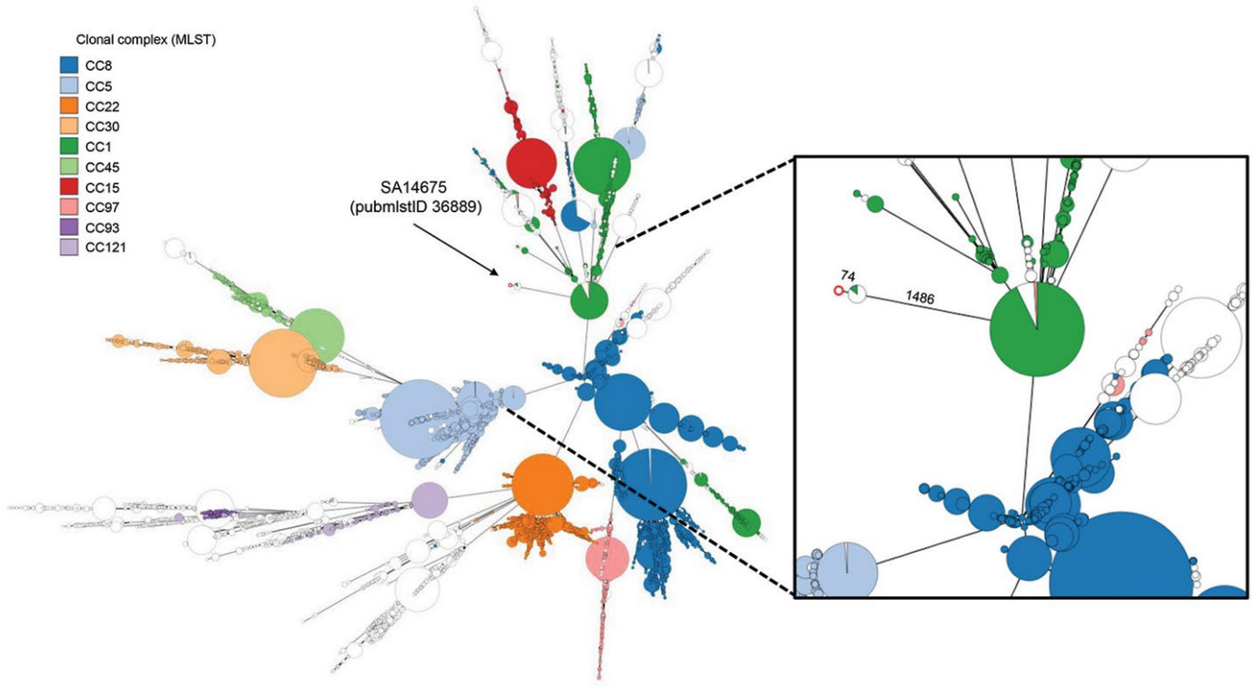

Appendix Figure 5. Genomic relationships of t991 isolates and 37,883 *S. aureus* strains isolated worldwide. The graph illustrates the genetic distance between the genomes included in the analysis. Grape tree was created on pubMLST site based on wgMLST data. Each node corresponds to a single CC. Node is proportional to genomes number. Strain SA14675 is marked by red circle and black arrow.
